# A programmable encapsulation system improves delivery of therapeutic bacteria in mice

**DOI:** 10.1038/s41587-022-01244-y

**Published:** 2022-03-17

**Authors:** Tetsuhiro Harimoto, Jaeseung Hahn, Yu-Yu Chen, Jongwon Im, Joanna Zhang, Nicholas Hou, Fangda Li, Courtney Coker, Kelsey Gray, Nicole Harr, Sreyan Chowdhury, Kelly Pu, Clare Nimura, Nicholas Arpaia, Kam W. Leong, Tal Danino

**Affiliations:** 1 Department of Biomedical Engineering, Columbia University, New York, NY USA; 2 Department of Microbiology and Immunology, Vagelos College of Physicians and Surgeons, Columbia University, New York, NY USA; 3 Herbert Irving Comprehensive Cancer Center, Columbia University, New York, NY USA; 4 Department of Systems Biology, Columbia University Medical Center, New York, NY USA; 5 Data Science Institute, Columbia University, New York, NY USA

**Keywords:** Synthetic biology, Cell delivery, Drug development, Drug delivery

## Abstract

Living bacteria therapies have been proposed as an alternative approach to treating a broad array of cancers. In this study, we developed a genetically encoded microbial encapsulation system with tunable and dynamic expression of surface capsular polysaccharides that enhances systemic delivery. Based on a small RNA screen of capsular biosynthesis pathways, we constructed inducible synthetic gene circuits that regulate bacterial encapsulation in *Escherichia coli* Nissle 1917. These bacteria are capable of temporarily evading immune attack, whereas subsequent loss of encapsulation results in effective clearance in vivo. This dynamic delivery strategy enabled a ten-fold increase in maximum tolerated dose of bacteria and improved anti-tumor efficacy in murine models of cancer. Furthermore, in situ encapsulation increased the fraction of microbial translocation among mouse tumors, leading to efficacy in distal tumors. The programmable encapsulation system promises to enhance the therapeutic utility of living engineered bacteria for cancer.

## Abstract (web summary)

Transient capsule induction allows engineered bacteria to evade initial immune surveillance in a colorectal cancer model.

## Main

The microbiome plays many functional roles in human health and subsequently has led to converging interests in the use of live bacteria to treat disease^1,2^. Because microbes can be engineered as intelligent living medicines that sense and respond to environments, they can colonize niches in the gastrointestinal tract^3,4^, mouth^5^, skin^6^, lung^7^ and tumors^8,9^ and locally deliver therapeutics. However, host toxicity from live bacteria has been shown to limit tolerated dose and efficacy, in some cases leading to termination of clinical trials^10–13^. Moreover, unlike conventional drug carriers, the unique abilities of bacteria to continuously proliferate, translocate and deliver therapeutic payloads in cancerous tissue necessitates robust and temporal control of bacterial pharmacokinetics in vivo.

One approach to circumvent immunogenicity and toxicity of living bacterial therapy is the generation of genetic knockouts of immunogenic bacterial surface antigens, such as lipopolysaccharide (LPS). But this strategy can result in permanent strain attenuation and reduced colonization, as seen in clinical trials of bacteria cancer therapy^11,14,15^. Surface modulation has been widely used in cloaking drug delivery vehicles^16^, and, therefore, an alternative strategy is the synthetic coating of microbial surfaces with molecules, such as alginate^17,18^, chitosan^17^, polydopamine^19^, lipids^20–22^ and nanoparticles^23^. These one-time, static modifications of bacteria do not allow in situ modulation and can lead to uncontrolled growth, off-target tissue toxicity or compromised cellular function, resulting in reduced efficacy. Thus, enhancing bacterial delivery without compromising safety is a central challenge for clinical translation of live microbial therapies in cancer.

**Fig. 1:**
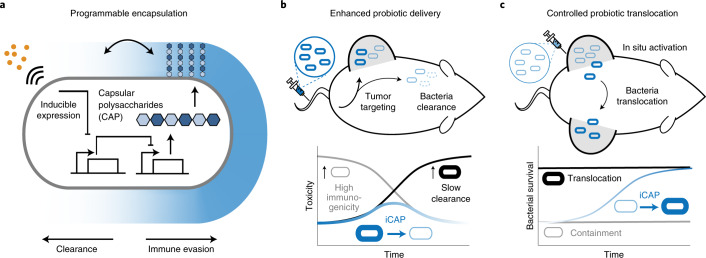
Programmable CAP system for control over bacterial encapsulation and in vivo delivery profiles. **a**, We engineered the biosynthetic pathway of bacterial CAP for tunable and dynamic surface modulation of the probiotic *E. coli* Nissle 1917 with synthetic gene circuits. This approach enables increased CAP levels upon induction to control immune evasion and clearance. **b**, The programmable CAP system enhances systemic delivery of bacteria by transiently expressing CAP. Non-CAP bacteria (thin gray cells) elicit toxicity by exposing the immunogenic bacterial surface, and permanently CAP-expressing bacteria (thick black cells) lead to overgrowth. The iCAP (blue cells) system enables transient encapsulation of bacteria, thus reducing initial inflammation while effectively clearing bacteria over time. **c**, The CAP system controls bacterial translocation among tumors. The iCAP system allows in situ activation of CAP in one tumor, which results in inducible bacteria translocation to distal, uncolonized tumors.

Here we present a tunable microbial surface engineering strategy using synthetic gene circuits to dynamically control bacterial interactions with their surrounding environment. We focused on bacterial surface capsular polysaccharide (CAP), a natural extracellular biopolymer that coats the extracellular membrane and protects microbes from a variety of environmental conditions. In the human body, CAP promotes bacterial survival and colonization by shielding microbes from various immune factors, such as complement opsonins and phagocytes^24^. Inspired by its natural ability to protect bacteria, we applied a synthetic biology approach to genetically engineer CAP biosynthesis for enhanced microbial delivery in vivo. Specifically, we constructed a programmable CAP expression system that regulates the bacterial surface with an external inducer, thereby modulating bacterial interaction with antimicrobials, bacteriophage, acidity and host immunity. This design allows precise control over bacterial immunogenicity and survivability in vivo, enabling increased dosing and in situ trafficking to maximize therapeutic efficacy and safety (Fig. 1).

### Small RNA knockdown screen identifies regulators of CAP synthesis

**Fig. 2:**
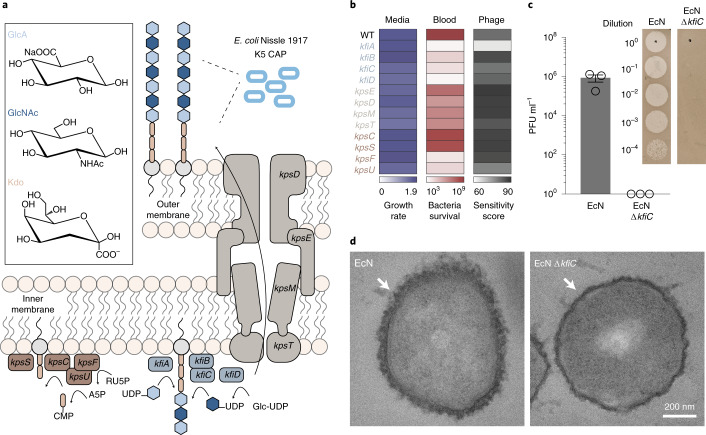
sRNA knockdown screen identifies key genes in CAP biosynthesis. **a**, Schematic of K5 CAP biosynthesis in EcN. CAP is composed of an alternating polymer chain of GlcA and GlcNAc connected to a poly-Kdo linker. Subsequently, CAP is transported from the inner bacteria membrane to the outer membrane. **b**, Quantification of microbial growth parameters of EcN KD strains in nutrient-rich media (LB), blood or phage-containing media. Growth rate denotes maximum specific growth rate (h^−1^) obtained by fitting growth curve to measured OD_600_ over time. Blood viability is defined as bacterial CFU ml^−1^ after 6-hour incubation in human blood. Phage sensitivity is calculated by area under the curve of bacterial turbidity over 6 hours of incubation with LB media containing ΦK1-5. **c**, Phage sensitivity of EcN and EcN Δ*kfiC*. Plaque-forming assay demonstrates complete absence of infection and lysis in EcN Δ*kfiC*. The representative images show difference between serially diluted PFU of bacteria with and without CAP. Error bars represent s.e.m. over three independent samples. **d**, TEM images showing CAP encapsulation of the cellular outer surface. *kfiC* KO results in the absence of CAP nanostructure on the cell surface of EcN Δ*kfiC*. White arrows indicate cell surface. WT, wild-type.

Because various bacteria have been used for therapeutic applications, we compared immunogenicity and viability of several *E. coli* and *Salmonella typhimurium* strains. Here, *E. coli* Nissle 1917 (EcN), a probiotic strain with favorable clinical profiles^25^, demonstrated high viability in human whole blood. When injected into mice, EcN induced the least immunogenic response in the blood (Supplementary Fig. 1). Because the K5-type CAP of EcN has been shown to alter interaction with host immune systems^26–29^, we chose to genetically modify its biosynthetic pathway^30,31^. K5-type CAP produced from EcN, also known as heparosan, is composed of a polymer chain of alternating β-D-glucuronic acid (GlcA) and N‐acetyl‐α‐D‐glucosamine (GlcNAc), attached to 3-deoxy-D-manno-oct-2-ulosonic acid (Kdo) linker (Fig. 2a). Glycotransferases of *kfiABCD* genes polymerize alternating GlcA and GlcNAc subunits. *kpsCSFU* genes encode proteins responsible for synthesis of the poly-Kdo linker on the terminal lipid, and *kpsEDMT* genes encode for transporters of CAP to the cellular surface. Although individual functions of the CAP genes have been investigated, engineering tunable and dynamic control of this system remains unexplored.

We sought to identify key CAP genes capable of altering response to antibacterial factors encountered during therapeutic delivery. To do so, we generated a library of knockdown (KD) strains using synthetic small RNAs (sRNAs) that reduce expression of *kfi* and *kps* genes via complementary binding to mRNAs^32^. To initially assess the effect of downregulating each gene, we screened the growth of KD strains in (1) nutrient-rich media, (2) human whole blood and (3) CAP-targeting phage. Growth in nutrient-rich media showed little variation in maximum specific growth rates (µ_m_) from the wild-type EcN strain (expressing CAP) (Fig. 2b), suggesting that the downregulation of the targeted genes in the CAP biosynthetic pathway does not greatly affect the fitness of EcN in the absence of environmental threats. However, we observed substantially reduced viability of KD strains compared to EcN after incubation in whole blood for 0.5 hours (Supplementary Fig. 2). After a 6-hour incubation in whole blood, KD strains in CAP synthesis (*kfi* genes and *kpsFU*) exhibited lower viability than KD strains in CAP transport (*kpsEDMT*) (Fig. 2b). To assess whether each gene KD causes a complete or partial loss of CAP in KD strains, we used lytic bacteriophage ΦK1-5 that specifically binds to heparosan of EcN^27,29^. Bacteria that express residual levels of heparosan CAP are susceptible to this phage, but complete loss of CAP confers immunity. We quantified phage sensitivity of each strain by measuring area under the curve of the phage-inoculated growth curve^33^ and observed that sRNA KD of most CAP genes did not alter phage sensitivity compared to control EcN (Fig. 2b), suggesting that some level of CAP is still present in KD strains. To abrogate the effect of residual CAP gene expression, we next constructed a library of knockout (KO) strains by deleting CAP synthesis genes (*kfiABCD*) from the EcN genome using the lambda red recombineering system. All *kfi* KO strains resulted in complete phage immunity (Supplementary Fig. 2), supporting loss of CAP expression. Notably, KO strains demonstrated increased blood sensitivity, indicating that CAP levels can be genetically tuned to alter sensitivities to antibacterial factors.

On the basis of the above results, we chose to further characterize *kfiC*, a well-studied gene that encodes a glycotransferase of GlcNAc. KfiC plays a central role in the production of heparosan by forming a membrane-associated complex with KfiA and KfiB, and the loss of this essential glycotransferase results in abolished CAP production^29,34,35^. Our results showed that (1) downregulation of *kfiC* via sRNA KD sensitized bacteria in blood, supporting its key role in regulating bacterial protection, and that (2) deletion of *kfiC* resulted in the highest enhancement in blood sensitivity, indicating that the level of protection can be altered by controlling gene expression. To confirm loss of CAP from the bacterial surface, we characterized surface properties of EcN Δ*kfiC* strain. Phage plaque formation assay confirmed complete immunity against ΦK1-5 (Fig. 2c). We also purified and detected bacterial polysaccharides using sodium dodecyl sulfate–polyacrylamide gel electrophoresis (SDS–PAGE), followed by CAP staining with alcian blue. Compared to EcN that produced strong staining with alcian blue at ~180 kDa, EcN Δ*kfiC* produced no visible band (Supplementary Fig. 3). We next characterized the morphological changes in bacterial surface using transmission electron microscopy (TEM) with ruthenium red staining. CAP was visible as an ~80-nm-thick layer of polysaccharides coating outside of the cellular membrane. In contrast, EcN Δ*kfiC* had diminished size of the polysaccharide layer at ~40 nm (Fig. 2d and Supplementary Fig. 3). We then investigated the capability of CAP to protect cells from a wide range of antimicrobial factors. In addition to the modified sensitivity to human whole blood and bacteriophages, EcN Δ*kfiC* demonstrated a significant reduction in cellular protection against panels of antibiotics (spectinomycin, ampicillin, gentamicin, kanamycin and streptomycin) and extreme acids (pH 2.5, LB media adjusted with hydrochloric acid) compared to EcN (Supplementary Fig. 4). Finally, we evaluated the general applicability of the approach in other CAP systems. We deleted homologous genes in different *E. coli* strains expressing K1 and K5 CAP (*neuC* and *kfiC*, respectively) and showed alteration in environmental sensitivity (Supplementary Fig. 5). Together, these results demonstrate that loss of CAP modifies cellular surface structure and protection against antimicrobial factors.

### Construction of tunable and reversible CAP

**Fig. 3:**
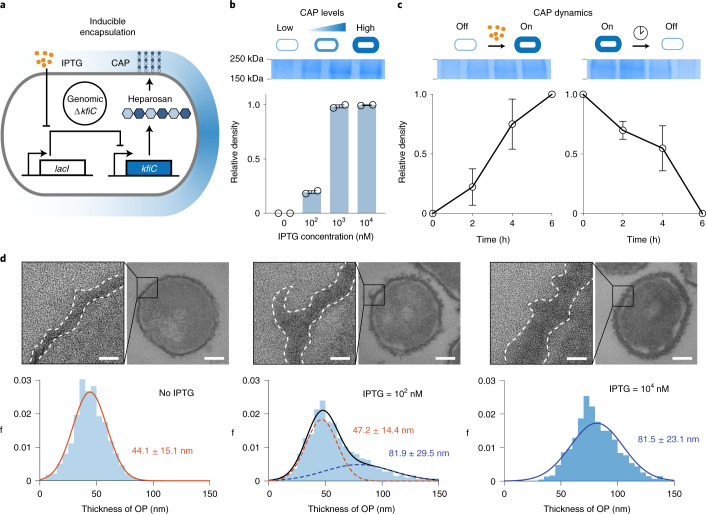
Design and characterization of the iCAP system. **a**, Inducible gene circuit diagram whereby the *kfiC* gene was cloned under the control of a *lac* promoter to allow inducible CAP expression via the small molecule IPTG. **b**, SDS–PAGE gel stained with alcian blue showed elevating levels of CAP production corresponding to the IPTG concentration (top). The densitometric analysis of CAP bands demonstrated that CAP production reaches maximum at approximately 1 µM IPTG (bottom). **c**, SDS–PAGE gels and densitometric analysis show CAP kinetics upon induction (left) and decay (right). For **b** and **c**, source data are provided as a Source Data file. **d**, Ruthenium-red-stained TEM images showing change in CAP in titrating IPTG concentration. Histograms reveal shift in cellular outer layer thickness as IPTG concentration increases. Insets show representative images of bacteria and zoomed outer surface structure. Dotted lines indicate inner and outer (white) perimeters of CAP. Scale bars, 40 nm (left) or 200 nm (right) in each inset. Five cells from each group were analyzed to generate the histograms. All error bars represent s.e.m. over two independent samples. OP, outer polysaccharide﻿. Source data

We next constructed a programmable CAP system that can sense and respond to induction stimuli and modulate cell surface properties. We cloned *kfiC* under the control of the *lac* promoter, which can be activated with the small molecule inducer isopropyl-b-D-thiogalactopyranoside (IPTG) (Fig. 3a). We built a small library of plasmids with various copy numbers of *kfiC* and tested combinations with *lacI* expressed from the EcN genome or a plasmid to optimize for tight regulation of CAP production. EcN Δ*kfiC* transformed with the low (sc101 origin) copy number plasmid exhibited complete immunity against ΦK1-5 (Supplementary Fig. 6), indicating tight repression at the basal level. Induction with IPTG rescued the phage sensitivity, confirming inducible modulation of CAP on cellular surface.

We examined the tunability of this inducible CAP (iCAP) system by characterizing multiple induction conditions. SDS–PAGE showed increase in CAP production from EcN carrying the iCAP system (EcN iCAP) when incubated with elevating levels of IPTG (Fig. 3b). Co-incubation with ΦK1-5 also showed decreasing viability of EcN iCAP with elevating levels of IPTG (Supplementary Fig. 6). We next used TEM to investigate the effect of the iCAP system on cell surface morphology (Fig. 3d). Increasing levels of IPTG shifted the mean bacterial membrane thickness from 44 nm to 81 nm, confirming the tunable capability of the system. Intermediate iCAP activation at 100 nM revealed a bimodal distribution of the membrane thickness, suggesting that *kfiC* regulates the production level, but not the length, of the polysaccharide polymers. This result agreed with SDS–PAGE data that showed no difference in migration of the CAP band depending on IPTG concentration (Fig. 3b).

We subsequently evaluated the dynamics of production and recovery of the iCAP system using a similar approach. Upon addition of IPTG, elevated CAP production was observed over time on SDS–PAGE, reaching near-maximum levels by 6 hours (Fig. 3c). Similarly, removal of IPTG resulted in gradual decrease in CAP until complete repression by 6 hours. We also tested iCAP dynamics via co-incubation with ΦK1-5. Although uninduced EcN iCAP grew, induction with IPTG at the start of co-incubation resulted in a rapid lysis event at 3.5 hours (Supplementary Fig. 6), demonstrating delayed CAP production with similar kinetics observed in SDS–PAGE. Collectively, these data highlight the programmable capability of CAP modulation on the bacterial surface.

### Programmable protection from host immunity

**Fig. 4:**
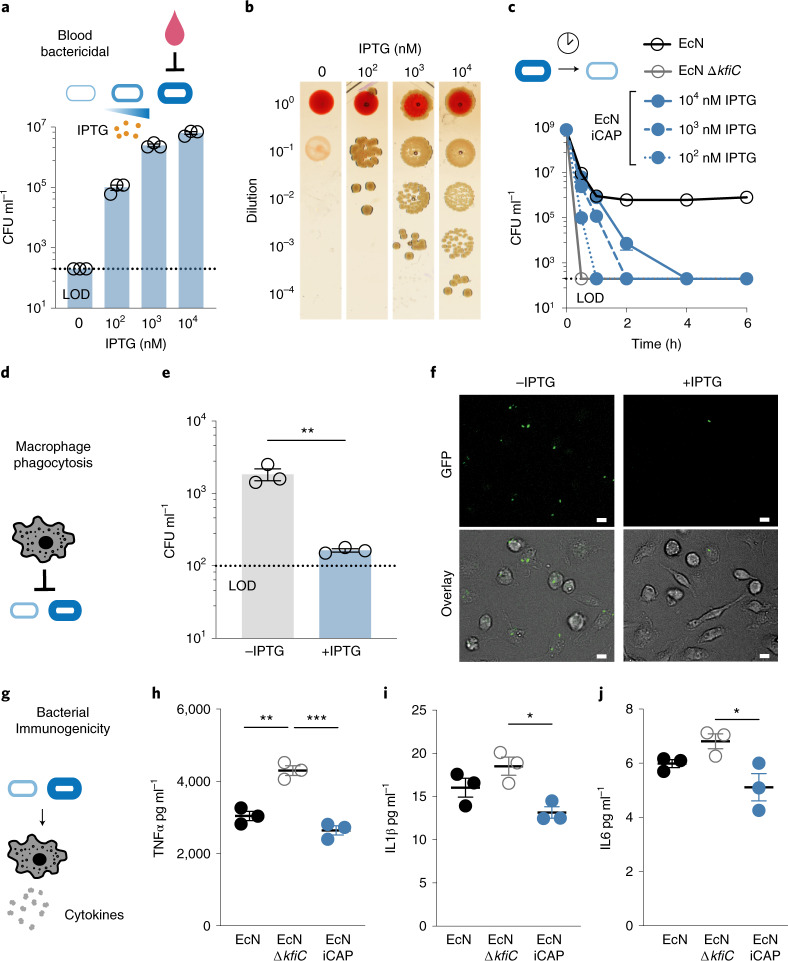
Tunable interaction of the CAP system with host immune factors. **a**, Bacteria were encapsulated using the iCAP system and exposed to human whole blood to test CAP-mediated protection. Elevating levels of CAP activation with IPTG enabled a corresponding increase in bacterial survival in human whole blood. Bacteria were pre-induced with IPTG before blood exposure. **b**, Representative images of bacteria spotted on LB agar plate after 1-hour incubation in human whole blood (right). **c**, Survival kinetics using varying levels of IPTG induction before incubation with human whole blood. 10^2^, 10^3^ or 10^4^ nM IPTG were added to the bacteria overnight culture to pre-induce the iCAP system. **d**, Induced or un-induced iCAP bacteria were co-cultured with BMDMs to test CAP-mediated protection from phagocytosis. **e**, BMDMs were lysed after incubating with bacteria for 30 minutes to enumerate phagocytosed bacteria (**P = 0.007, two-sided unpaired *t*-test). **f**, Representative fluorescence microscopy images showing bacteria (GFP, top) in phagocytes (bright-field overlayed with GFP, bottom). Scale bars, 10 µm. **g**, Human THP-1 cells were co-incubated with EcN, EcN *∆kfiC* or EcN iCAP (pre-induced with 10 µM IPTG) to test for immunogenicity. **h**–**j**, Cytokine levels in the culture media were measured using Luminex multiplex assay including TNFα (**P = 0.001 and ***P = 0.0002, respectively, at 24 hours after incubation), IL1β (*P = 0.017 at 4 hours after incubation) and IL6 (*P = 0.029 at 4 hours after incubation). All error bars represent s.e.m. over three independent samples, and statistical analyses were performed using one-way ANOVA with Tukey’s multiple comparison test. LOD at 2 × 10^2^ CFU ml^−1^ (**b** and **c**) and 1 × 10^2^ CFU ml^−1^ (**e**).

To build toward use of the iCAP system for cancer applications in vivo, we first tested the ability to control bacterial viability in human whole blood containing functional host bactericidal factors in vitro. Upon IPTG induction, we observed increased EcN iCAP survival compared to uninduced control (Fig. 4a,b). Increasing IPTG levels improved bacterial survival over a range of at least ~10^5^-fold, highlighting the tunable capability of the system. Because iCAP deactivation was observed after removing the inducer, we tracked bacterial survival over time after transiently activating EcN iCAP at varying IPTG concentrations. We were able to modulate the rate of bacterial clearance from blood by titrating levels of IPTG (Fig. 4c). Wild-type EcN persisted for more than 6 hours, whereas EcN Δ*kfiC* quickly decreased to the levels under the limit of detection (LOD, ~10^2^ colony-forming units (CFU) per milliliter) within the first 0.5 hours. A protective role of CAP in mouse whole blood was also observed (Supplementary Fig. 7).

To investigate the effect of the programmable CAP system on bacterial interaction with individual immune factors within whole blood, we assessed how CAP alterations modulated macrophage-mediated phagocytosis and complement-mediated killing. To study phagocytosis, we incubated EcN with murine bone-marrow-derived macrophages (BMDMs) (Fig. 4d). iCAP activation before co-incubation with macrophages resulted in reduction in uptake of bacteria within macrophages compared to basal control (Supplementary Fig. 8), demonstrating controllable protection from cellular immune recognition. Bacterial colony counting of macrophage lysates and fluorescence microscopy imaging confirmed ~10-fold less phagocytosis with bacteria induced with IPTG compared to uninduced control (Fig. 4e,f). To assess the inflammatory response by phagocytes, we co-cultured EcN with THP-1 human monocytic cells and measured levels of various cytokines produced in response to microbial detection (Fig. 4g). Absence of CAP increased levels of many inflammatory cytokines (Fig. 4h–j and Supplementary Fig. 9), indicating the ability of CAP to mask microbial recognition from the immune system. Activated EcN iCAP was able to decrease inflammatory responses to similar levels as EcN. To study protection against circulating host antimicrobials, such as the complement system, we exposed EcN to human plasma. Presence of CAP improved bacterial survival by at least ~10^5^-fold (Supplementary Fig. 10), demonstrating that CAP protects bacteria from soluble host bactericidal factors. Together, these findings suggest the potential utility of the programmable CAP system to modulate a multitude of host–microbe interactions in vivo.

### Transient CAP improves engineered probiotic therapy in vivo

**Fig. 5:**
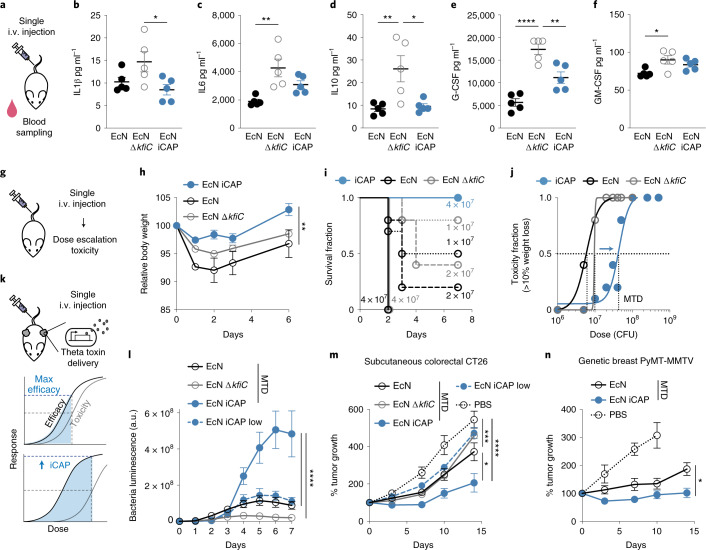
Transient CAP activation improves systemic bacterial delivery and efficacy in vivo. **a**, Host response was evaluated after bacterial administration in mice. EcN iCAP was pre-induced with 10 µM IPTG and allowed to gradually attenuate CAP over time. **b**–**f**, Serum cytokine levels after 5 × 10^6^ CFU bacterial administration. IL1β (**b**, *P = 0.039 at 4 hours p.i.), IL6 (**c**, **P = 0.0037 at 4 hours p.i.), IL10 (**d**, **P = 0.0089 and *P = 0.014, respectively, at 24 hours p.i.), G-CSF (**e**, ****P < 0.0001 and **P < 0.01, respectively, at 24 hours p.i.) and GM-CSF (**f**, *P = 0.019 at 4 hours p.i.) were measured. **g**, Toxicity was evaluated by bacterial administration with elevating doses. **h**, Change in animal body weight after i.v. bacterial administration at 5 × 10^6^ CFU (**P = 0.004; *n* = 10, 5 and 5 mice for EcN iCAP, EcN and EcN Δ*kfiC* groups, respectively). **i**, Survival curve (>15% body weight reduction; *n* ≥ 5 mice per group) after bacterial administration at 1~7 × 10^7^ CFU. **j**, Dose–toxicity curve with MTD = 4.4 × 10^7^ CFU, 5.8 × 10^6^ CFU and 9.6 × 10^6^ CFU for EcN iCAP, EcN and EcN Δ*kfiC*, respectively. MTD was calculated based on TD_50_ (>10% body weight drops p.i.; non-linear regression with least squares fit; *n* ≥ 5 per group). **k**, Mice bearing tumors were intravenously injected with EcN MTD, EcN Δ*kfiC* MTD and EcN iCAP MTD (pre-induced with 10 µM IPTG) or EcN iCAP low (pre-induced with 10 µM IPTG) at 5 × 10^6^, 1 × 10^7^, 5 × 10^7^ or 5 × 10^6^ CFU, respectively. Bacteria were engineered to produce TT. **l**, Bacterial growth trajectories in subcutaneous CT26 tumors measured by bacterial luminescence (****P < 0.0001; *n* = 14, 13, 9 and 13 tumors for EcN MTD, EcN Δ*kfiC* MTD, EcN iCAP MTD and EcN iCAP low groups, respectively). Luminescence values are normalized to basal luminescence of individual strains. **m**, **n**, Therapeutic efficacy measured by relative tumor size over time in a syngeneic CT26 model (**m**; ****P < 0.0001, ***P = 0.0008, **P = 0.003; *n* = 14, 13, 9, 13 and 11 tumors for EcN MTD, EcN Δ*kfiC* MTD, EcN iCAP MTD, EcN iCAP low and PBS groups, respectively) and in a genetically engineered spontaneous breast cancer MMTV-PyMT mouse model (**n**; *P = 0.0197; *n* = 15, 15 and 9 tumors for EcN MTD, EcN iCAP MTD and PBS groups, respectively). MMTV-PyMT tumors were measured by calipering three orthotopic regions in mammary glands (upper left, upper right and bottom). Mice in PBS groups reached study endpoint 10 days p.i. Statistical analyses were performed using one-way ANOVA (**b**–**f**) and two-way ANOVA (**h**, **i**–**n**) with Tukey’s multiple comparison test. Bacteria were engineered to produce TT (**l**–**n**). All error bars represent s.e.m. over three independent samples unless otherwise noted. All ‘*n*’ denotes number of biological replicates. a.u., arbitrary units; GM-CSF, granulocyte–macrophage colony-stimulating factor; p.i., post injection.

Intravenous (i.v.) delivery of bacteria allows access to various disease sites in the body; however, systemic delivery of bacteria remains challenging because (1) rapid clearance by the host immune system requires increased dosing, whereas (2) failure in bacteria clearance can lead to bacteremia and sepsis. Because the iCAP system allowed temporal control over bacterial protection and immunogenicity, we sought to improve bacterial delivery by initially protecting bacteria during the delivery stage via CAP production and subsequently allowing CAP decay to clear them and ensure safety. To study the protective role of CAP in vivo, we first characterized probiotic bioavailability and host health in mouse models. Upon i.v. administration of EcN Δ*kfiC*, viable bacteria in blood circulation quickly dropped below the LOD (200 CFU ml^−1^). In contrast, EcN remained detectable during the first 4 hours (Supplementary Fig. 11), demonstrating the protective function of CAP in vivo. To examine the host response to encapsulated (that is, wild-type) versus unencapsulated (that is, Δ*kfiC*) EcN, we measured levels of serum cytokines (Fig. 5a). We detected lower levels of acute inflammatory cytokines from mice injected with EcN compared to EcN Δ*kfiC* (Fig. 5b–f and Supplementary Fig. 11), similarly to the decreased response observed in our in vitro assay. To test the effect of initial encapsulation by EcN iCAP, we administered the strain pre-induced with 10 µM IPTG and compared serum cytokine levels. EcN iCAP generally induced less inflammatory response than EcN Δ*kfiC*, supporting successful encapsulation in vivo. We noted that EcN iCAP appeared to elicit intermediate production of a subset of cytokines (such as interleukin 6 (IL6) and granulocyte colony-stimulating factor (G-CSF)) in between EcN and EcN Δ*kfiC*, and the contribution of the transient encapsulation system to this response warrants future investigation. To study long-term inflammation, we tracked white blood cell count over time. In contrast to the acute cytokine responses, we detected low neutrophil counts initially that increased over 1 week after injection with the bacteria (Supplementary Fig. 11). EcN administration resulted in a higher level of neutrophil counts compared to EcN iCAP, suggesting that the persistence of CAP-expressing EcN poses a risk of systemic inflammation and toxicity. Thus, although CAP can improve bioavailability and lower initial immunogenicity, static protection might lead to bacterial persistence and inflammatory response, posing toxicity risks.

We hypothesized that transient activation of the iCAP system could enhance bacterial delivery properties, such as maximum tolerated dose (MTD), host toxicity and biodistribution. Inducing CAP expression before injection would increase bioavailability and mask cytokine induction, and loss of CAP in the absence of the inducer in vivo would allow for effective clearance of bacteria and minimize long-term immune responses. To test this strategy, we first pre-induced EcN iCAP with 10 µM IPTG, intravenously administered escalating doses of the bacteria and then assessed host health and determined MTD^36,37^(Fig. 5g). At 5 × 10^6^ CFU, EcN iCAP caused a smaller decrease in body weight compared to EcN and EcN Δ*kfiC* with static cellular surface (that is, with or without CAP, respectively) (Fig. 5h). Notably, EcN iCAP reduced toxicity compared to EcN and EcN ∆*kfiC* at higher doses: EcN and EcN Δ*kfiC* caused severe endpoint toxicity (death or >15% loss of weight) to mice treated with doses above 1 × 10^7^ CFU within 2 days, whereas no mice showed severe toxicity after injection of pre-induced EcN iCAP at the same doses (Fig. 5i). Based on these data, we computed a dose–toxicity curve and demonstrated that transiently induced EcN iCAP results in ~10-fold-higher MTD compared to EcN and EcN Δ*kfiC* (Fig. 5j). To further study safety, we simulated a severe toxicity scenario by inducing sepsis by intraperitoneal injection of bacteria^38^. At both high and low doses (10^7^ and 10^6^ CFU, respectively), we consistently observed improved safety for EcN iCAP compared to EcN and EcN Δ*kfiC* (Supplementary Fig. 12). Finally, to study bacteria biodistribution, we administered all groups of bacteria at a matched dose of 5 × 10^6^ CFU via i.v. injection. Approximately 10-fold less EcN and EcN iCAP were found in peripheral organs (liver and spleen) compared to EcN Δ*kfiC* (Supplementary Fig. 13), indicating that initial induction of EcN iCAP was sufficient to provide protection from the mononuclear phagocyte system.

Because systemic bacterial delivery has been extensively used for cancer therapy, we next tested whether the programmable CAP system can improve anti-tumor efficacy by permitting higher doses (Fig. 5k). To engineer bacteria to deliver anti-tumor payloads, we cloned a gene encoding theta toxin (TT), a pore-forming toxin that was previously shown to be effective as a bacterial cancer therapy^39^, in a high-copy-number plasmid (pUC) with a stabilization mechanism for in vivo applications (Axe/Txe system^40^). In a syngeneic CT26 colorectal cancer model, we intravenously administered engineered EcN at the corresponding MTD of each strain (EcN, EcN Δ*kfiC* and EcN iCAP at 5 × 10^6^, 1 × 10^7^ and 5 × 10^7^ CFU, respectively), along with a low dose of EcN iCAP at 5 × 10^6^ CFU to match the MTD of EcN. Over the following days, bacterial accumulation in tumors was observed by luminescence. Here, EcN iCAP MTD showed significantly higher signals in tumors compared to all other groups (Fig. 5l). After bacterial administration, we observed that mice treated with EcN MTD, EcN Δ*kfiC* MTD and low-dose EcN iCAP exhibited modest tumor growth suppression compared to the untreated group over 14 days. By contrast, single administration of EcN iCAP MTD resulted in significant tumor growth suppression by ~400% compared to the untreated group (Fig. 5m). Although increased MTD enabled by transient activation of the iCAP system improved therapeutic efficacy, the body weight of animals in MTD groups remained similar (Supplementary Fig. 14). We next compared the efficacy of TT-producing EcN and EcN iCAP at MTD in a genetically engineered spontaneous breast cancer model (MMTV-PyMT). EcN iCAP MTD resulted in improved tumor growth suppression by ~100% compared to EcN MTD over 14 days (Fig. 5n). Consistently, we observed higher bacterial luminescence signal in tumor from EcN iCAP compared to EcN, whereas body weight remained similar between the two treatment groups (Supplementary Fig. 15). To further explore the role of CAP on bacterial delivery to tumors in vivo, we built a mathematical bacterial pharmacokinetics model. Our simulations suggested that transient protection of bacteria using the iCAP system could improve tumor specificity by minimizing persistence in peripheral organs (that is, blood and liver), supporting our experimental observations (Supplementary Fig. 16). As a result, this approach allows for elevated bacterial doses, which leads to increased tumor accumulation upon injection. Lastly, we tested whether EcN iCAP had lost CAP in tumors. We injected EcN, EcN Δ*kfiC* and EcN iCAP (pre-induced with IPTG) at a dose of 5 × 10^6^ CFU via i.v. injection, isolated intratumoral bacteria after 2 days and grew the bacteria in LB media containing ΦK1-5. EcN iCAP grew in the presence of ΦK1-5, indicating the loss of CAP in the tumor (Supplementary Fig. 17). Taken together, the iCAP system enables increased tolerable bacterial doses and improved therapeutic efficacy.

### In situ CAP activation translocates EcN to distal tumors

**Fig. 6:**
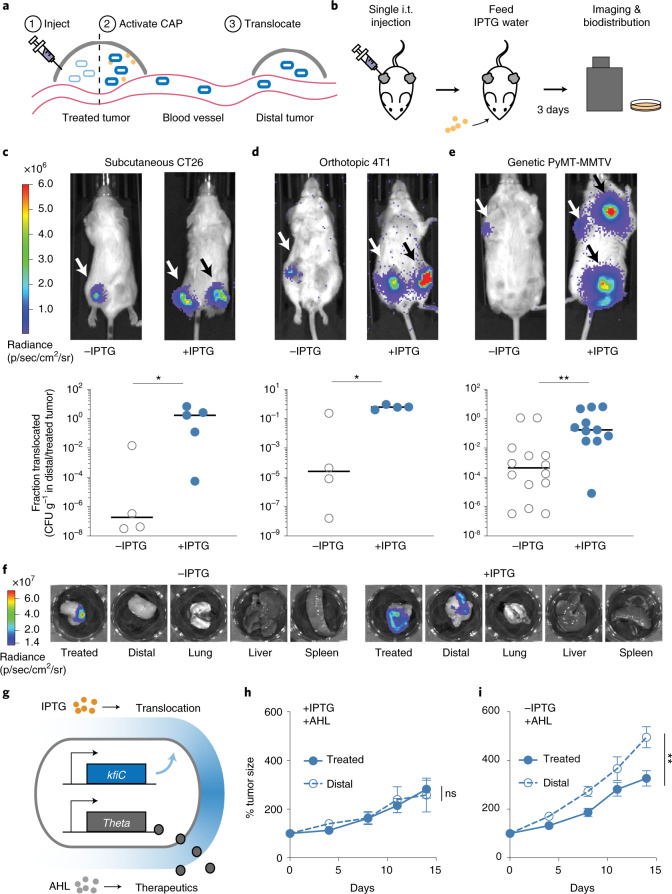
In situ activation of the CAP enables bacterial translocation and drug delivery to distal tumors. **a**, Schematic of iCAP-mediated bacterial translocation. EcN iCAP is injected into one tumor. iCAP activation enables bacteria translocation to distal tumors. **b**, Mice harboring multiple tumors are injected with EcN iCAP into a single tumor (treated tumor). Mice are fed 10 mM IPTG water to activate iCAP in situ. To quantify bacterial biodistribution, mice were imaged daily for bacterial bioluminescence, and organs were harvested and bacterial colonies were counted after 3 days. **c**–**e**, Inducible translocation of EcN iCAP to distal tumors in CT26 syngeneic (**c**), 4T1 orthotopic (**d**) and MMTV-PyMT genetically engineered (**e**) mouse tumor models. Representative IVIS images showing bacterial translocation in vivo. White arrows indicate location of bacterial injection. Black arrows indicate location of bacterial translocation. Translocation is quantified by fraction of bacteria found in distal tumor compared to treated tumor. Bacteria number is measured by performing biodistribution for CFU g^−1^ enumeration for CT26 (*P = 0.032; *n* = 4 and 5 tumors for −IPTG and +IPTG groups, respectively), 4T1 (*P = 0.029; *n* = 4 tumors for both −IPTG and +IPTG groups) and MMTV-PyMT (**P = 0.003; *n* = 14 and 11 tumors for −IPTG and +IPTG groups, respectively) models. All error bars represent median, and statistical analyses were performed using a two-sided Mann–Whitney test. **f**, Representative images of ex vivo organ images taken with IVIS showing bacterial tumor translocation in 4T1 orthotopic mouse model. **g**, Schematics of engineered EcN capable of inducible translocation and therapeutic expression. Translocation and therapeutic production are externally controlled by IPTG and AHL, respectively. **h**–**i**, Therapeutic efficacy in treated and distal CT26 tumors measured by relative tumor growth over time. All bacteria were engineered to produce TT. Bacteria were injected into a single treated tumor. Three days p.i., AHL was administered. Mice were fed with (**h**) or without (**i**) IPTG water, and tumor size was measured (NS *P* = 0.83 and **P = 0.004, respectively; two-way ANOVA, *n* = 6 and 5 for treated and distal tumors, respectively). All error bars represent s.e.m. All ‘*n*’ denotes number of biological replicates. NS, not significant; p.i., post injection.

Intratumoral (i.t.) bacteria injection has been used as a route of delivery in clinical settings owing to higher therapeutic efficacy, dose titration capability and improved safety profiles compared to systemic injection^41–45^. One unique capability of i.t. delivery is the translocation of bacteria from injected tumors to distal tumors^45^, potentiating an alternate route of safe bacterial delivery to inaccessible tumors. However, continuous translocation coupled with long-term survival of bacteria can pose a substantial safety concern; thus, in situ activation of translocation could allow for more optimal use of this phenomena. To model this hypothesis, we simulated i.t. delivery and showed that in situ induction of EcN iCAP within the tumor increases bacterial bioavailability in circulation and facilitates bacterial translocation to distal tumors (Fig. 6a and Supplementary Fig. 16). We then tested this strategy via i.t. injection of uninduced EcN iCAP (that is, without CAP) into a single tumor of mice harboring dual hind flank CT26 tumors (Fig. 6b). To activate the iCAP system in situ, mice were fed with water containing IPTG. After 3 days, we observed a marked increase in bacterial translocation to distal tumors compared to uninduced bacteria (Fig. 6c and Supplementary Fig. 18). Biodistribution data showed tumor translocation, and mice exhibited minor reductions in body weight (Supplementary Fig. 18). Tracing colonization kinetics using bioluminescent EcN confirmed the appearance of bacteria in distal tumor 1 day after IPTG administration (Supplementary Fig. 19). We next explored whether this bacterial trafficking approach can be generalized in multiple clinically relevant animal models. We tested orthotopic breast cancer (mammary fat pad 4T1) and MMTV-PyMT mouse models. Consistently, we observed increased bacterial translocation to distal tumors via in situ activation of iCAP in both tumor models (Fig. 6d–f and Supplementary Figs. 19, 20 and 21). Notably, i.t. injection of EcN iCAP into a single tumor in the MMTV-PyMT model resulted in microbial translocation to multiple distal tumors throughout the body after IPTG induction.

We next delivered therapeutics to tumors using engineered EcN expressing the anti-tumor TT payload. TT was cloned under the *luxI* promoter that is responsive to an inducer molecule acylated homoserine lactone (AHL) orthogonal to IPTG (Fig. 6g). After i.t. injection of therapeutic EcN into a single tumor in the CT26 dual-flank mouse model, translocation to uninjected tumors was controlled by feeding mice with or without IPTG water. Another group of mice was given i.t. injection of EcN iCAP without TT as non-therapeutic control. iCAP-mediated bacterial translocation was confirmed by bacterial bioluminescence (Supplementary Fig. 22). Subsequently, AHL was administered subcutaneously to induce TT expression, and tumor growth was monitored. Although PBS treatment allowed tumor growth of ~400%, a reduction in the growth of tumors was observed when they were directly injected with therapeutic EcN regardless of bacterial translocation. By contrast, therapeutic efficacy in distal (uninjected) tumors was observed only when the mice were fed with IPTG water followed by TT induction via subcutaneous injection of AHL (Fig. 6h,i and Supplementary Fig. 23), demonstrating successful therapeutic delivery to distal tumors using the iCAP-mediated bacterial translocation approach. Body weight recovered to baseline within a few days after administration for all conditions (Supplementary Fig. 24). Together, we provide a demonstration of controllably translocating therapeutic bacteria and using this strategy to treat distal tumors in vivo.

## Conclusion

We have demonstrated a synthetic biology approach for dynamic and tunable modulation of the bacterial surface in the context of in vivo therapeutic delivery. Taking advantage of the natural evolution of the CAP system to interface with a multitude of environments, we showed engineered bacterial interactions with host immunity, bacteriophage, antimicrobials and acidity. Although we have explored the modulation of CAP density via *kfiC* in this study, our sRNA screen showed that several genes differentially sensitized bacteria to immune factors in blood. Thus, additional genes identified in this study may also be used to control varied sensitivities or to independently alter bacterial sensitivity to various environmental factors. For example, *kfiB* and *kfiC* genes have been reported to differentially modulate bacterial interactions with epithelial cells^29^. We have also shown that this approach can be applied to other strains of *E. coli*. Because there exist over 80 distinct *E. coli* CAP systems^24,31^ and many more in other species, we envision CAP engineering to possess vast opportunities to controllably modulate microbial surface properties for therapeutic delivery.

Despite recent preclinical progress with bacterial therapies, dose-limiting toxicity has been a long-standing challenge, slowing efforts for clinical translation. Early works by William Coley in the 19th century observed tumor regression upon injections of a live bacterial cocktail^46^, but this approach was largely unsafe due to potential risks of infections and inflammatory side effects. More recently, several clinical trials using genetically attenuated bacterial strains observed dose-limiting toxicities. For example, systemic administration of attenuated *S. typhimurium* strain (VNP20009) at or higher than tolerable dose led to tumor colonization in less than 20% of patients and resulted in no objective regression in a phase 1 clinical trial^11^. Although the recent focus of synthetic biology has been the engineering of various therapeutic payloads to increase efficacy, strategies to improve bacterial delivery have been limited. We used an on-demand CAP system to temporarily protect probiotic EcN from immune clearance and demonstrated a ~10-fold increase in the systemically injectable tolerated dose in vivo. As a result, transient expression of CAP was able to safely enhance bacterial delivery and suppress rapidly growing syngeneic colorectal and breast tumors with a single i.v. administration. Given that humans are 250-fold more sensitive to endotoxins than mice^47^, we expect our results to have implications in the clinical translation of bacterial therapies. Further investigation on the safety of this approach for human patients is needed. For example, comprehensive analysis on inflammatory responses at escalating doses can provide insights toward establishing an MTD and account for differences from in vitro human and in vivo mouse results. We also noted that EcN iCAP appeared to result in slightly more weight loss initially compared to EcN at their respective MTD, and further investigation with a larger sample size is warranted. Leveraging the programmable nature of the genetic CAP system, we envision the use of biosensors to autonomously sense physiological condition in the future. Because the CAP system is orthogonal to other bacterial surface structures, such as LPS, phospholipids and flagellum^48^, synergistic combinations of these systems could further improve the programmability and immunogenicity of bacterial therapy. More broadly, tools for controlling bacterial growth, such as bacterial lysis, auxotrophy and antibiotics, can be combined with the CAP system to enhance safety^49–54^.

We further devised the CAP system to facilitate translocation of bacteria to distal tumors, demonstrating a delivery strategy with advantageous safety profiles. Comparing across different tumor types, we noted leakier translocation of bacteria in 4T1 and MMTV-PyMT models, suggesting that tumor type, location or vascularization might play a role in bacterial escape from the tumor microenvironment. Regardless, in situ activation of the iCAP system opens the possibility of using programmable translocation of therapeutic bacteria to inaccessible disease sites, including metastatic tumors. Transient in situ activation could allow for colonization of newly formed tumors with reduced accessibility. In addition, the ability to transiently increase circulating bacteria from colonized tumor might allow safe sampling of bacteria without the need for an invasive tumor biopsy, while preventing excessive leakage of bacteria that could cause bacteremia and systemic infection. More broadly, we envision that in situ control over bacterial CAP may be used to provide further safeguards during bacterial therapy, such as sensitization of bacteria with antibiotics and phage therapy^53–55^.

In addition to clear applications within bacteria cancer therapy, the utility of the programmable CAP system can be extended to other clinical settings. For example, we showed that CAP provides protection from acids that could potentially protect orally delivered probiotics during transit through the gastric environment and facilitate intestinal colonization^56^. Beyond delivery, altering surface immunogenicity may also be used therapeutically to modulate the host immune landscape in the tumors and gut^57,58^. The platform described here could also be used as a model system to study the role of CAP and other surface structures on pathogen colonization in the host environment^59,60^. Aside from model systems and clinical applications, the CAP system may provide a general platform for a programmable interface with various environments. As microbial deployment in various applications continues to advance, robust control over microbial interaction with complex surroundings will ensure safe and effective implementation of engineered microbes.

## Methods

### Bacterial strains and culturing

The host strain used in this study was EcN, which naturally expresses K5 CAP containing a genomically integrated erythromycin resistance *luxCDABE* cassette for bacterial bioluminescence tracking in vivo. For all strains used in this study, see Supplementary Table 1. All bacteria were grown with appropriate antibiotics selection (100 μg ml^−1^ of ampicillin, 50 μg ml^−1^ of kanamycin, 25 μg ml^−1^ of chloramphenicol and 50 µg ml^−1^ of erythromycin) in LB media (Sigma-Aldrich) at 225 r.p.m. or on LB agar plates containing 1.5% agar at 37 °C.

### Construction of plasmids and gene circuits

To construct a KD library, plasmids with sRNA targeting each gene of the CAP biosynthetic pathway were prepared using Gibson Assembly. The sRNA sequences were designed to be complementary and bind to the 24-nucleotide sequence of the target gene coding sequence spanning the ribosome binding site and the start codon^32,61^. A plasmid template was prepared by polymerase chain reaction (PCR) amplifying backbone (pTH05) using primers (pTH05_for and pTH05_rev), and the single-stranded DNA for sRNA against genes in CAP biosynthesis was inserted (*kfiA*, *kfiB*, *kfiD*, *kpsC*, *kpsS*, *kpsF*, *kpsU*, *kpsE*, *kpsD*, *kpsT* and *kpsM*) and transformed into Mach1 competent cells (Invitrogen). CAP gene circuits and the therapeutic plasmids were constructed in a similar manner. Genes of interest were obtained by synthesizing oligos or gBlock from IDT or PCR amplification (*kfiC* gene was obtained via colony PCR from EcN). Subsequently, plasmids were constructed using Gibson Assembly or using standard restriction digest and ligation cloning and transformed into Mach1 competent cells (Invitrogen).

### Construction of KO strains

EcN was transformed to carry lambda red helper plasmid (pKD46)^62^. Transformants were grown in 50 ml of LB at 30 °C with chloramphenicol to an optical density at 600 nm (OD_600_) of 0.4 and made electrocompetent by washing three times with ice-cold MilliQ water and concentrating 150-fold in 15% glycerol. Chloramphenicol resistance cassette was prepared by PCR with primers flanked by sequence within each target gene, followed by gel purification and resuspension in MilliQ water. Electroporation was performed using 50 µl of competent cells and 10–100 ng of DNA. Shocked cells were added to 1 ml of SOC, incubated at 30 °C for 1 hour with 20 µl of arabinose and incubated at 37 °C for 1 hour. Cells were then plated on LB plates with chloramphenicol and incubated in 37 °C overnight. Colonies were picked the next day to obtain KO strains, including Δ*kfiC* strain (EcN *ΔkfiC*).

### Characterization of CAP strains sensitivity to phages, antibiotics and acids

To perform plaque-forming assays, bacteria were plated onto LB agar plates to make a lawn and allowed to dry under fire. Then, 10 µl of serial diluted ΦK1-5 phage (I. Molineux, University of Texas, Austin) was spotted onto the plates and allowed to dry. Plates were incubated at 37 °C overnight and inspected the next day for PFU counting. Similar phage plaque-forming assays were performed for K1 and K5 type *E. coli* strains.

To assess bacterial growth in liquid culture, overnight cultures of EcN, EcN *ΔkfiC* or EcN iCAP strains were calibrated into OD_600_ of 1.0, and 100 µl of each was transferred into a 96-well plate (Corning). Next, 1 µl of 10^8^ PFU ΦK1-5 phage or antibiotics of indicated concentrations was added to each well. The samples were incubated at 37 °C with shaking in a Tecan plate reader, and the OD_600_ was measured every 20 minutes. For bacterial growth in low pH condition, LB media was adjusted to pH 2.5 using hydrochloric acid, and bacteria were incubated at 37 °C for 1 hour, followed by serial dilution and plating on an LB agar plate for CFU enumeration.

### Characterization of CAP using SDS–PAGE

CAP was purified via the chloroform–phenol extraction as previously described^63,64^. In brief, 3 ml of overnight bacteria cultures were harvested the next day and further subcultured in 50 ml of LB broth in the presence or absence of 0.1 M IPTG for indicated lengths of time. Bacteria concentrations were adjusted to the same level across samples via OD_600_ before centrifugation at 3,000*g*. Pellets were collected and resuspended in 150 µl of water. An equal amount of hot phenol (65 °C) was added, and the mixtures were vortexed vigorously. The mixtures were then incubated at 65 °C for 20 minutes, followed by chloroform extraction (400 µl) and centrifugation. The CAP was detected by alcian blue staining as previously reported^64–66^. In brief, after SDS–PAGE electrophoresis (4–20% gradient), the gel was fixed in fixing solution (25% ethanol, 10% acetic acid in water) for 15 minutes while shaking at room temperature. The gel was then incubated in alcian blue solution (0.125% alcian blue in 25% ethanol, 10% acetic acid in water) at room temperature for 2 hours while shaking before de-stained overnight in fixing solution. CAP was visualized as alcian blue stained bands on the resulting gel.

### Visualization of CAP using TEM

Bacteria were grown overnight in LB media with appropriate antibiotics before being processed for imaging. For EcN iCAP, a 1:100 dilution in LB with antibiotics was made the following day and grown in 37 °C shaker until OD_600_ = 0.1–0.4 (mid-log phase), and varying concentrations of IPTG were added for further incubation for 6 hours before being processed. The cultures were spun down at 300*g* for 10 minutes and embedded in 2% agarose. Each agarose gel fragment was cut into a cube with 2-mm edge and placed in a 1.5-ml centrifuge tube. The samples embedded in agarose were fixed and stained via protocols previously reported^67^. In brief, the samples were fixed with 2% paraformaldehyde and 2.5% glutaraldehyde in osmotically adjusted buffer (0.1 M sodium cacodylate, 0.9 M sucrose, 10 mM CaCl_2_, 10 mM MgCl_2_) with 0.075% ruthenium red and 75 mM lysine acetate for 20 minutes on ice. The samples were washed with osmotically adjusted buffer containing 0.075% ruthenium red twice and further fixed with 1% osmium tetroxide in osmotically adjusted buffer containing 0.075% ruthenium red for 1 hour on ice. The samples were washed three times in water with 5-minute incubation between each wash and dehydrated in increasing concentrations of ethanol (50%, 70% and 100%) on ice for 15 minutes per step. The samples were washed one more time in 100% ethanol and embedded in increasing concentrations of Spurr’s resin (33% and 66%) diluted in ethanol for 30 minutes per step and overnight in 100% Spurr’s resin. The samples were moved to fresh Spurr’s resin the next day and polymerized at 65 °C overnight before being sectioned using a Sorvall MT-2B Ultramicrotome to ~70 nm. The sample sections were placed on TEM grids (Ted Pella, 01800F) and stained using UranyLess (EMS). The sample grids were imaged using FEI Talos 200 TEM.

### TEM image processing and data analysis of polysaccharide layer

The image processing of TEM images was performed using ImageJ, and the data analysis was done using MATLAB. Due to low signal-to-noise ratio of the TEM images resulting from thinly sectioned bacteria samples stained using ruthenium red, Gaussian blur was used to reduce the noise and help determine the boundary of the polysaccharide layer. The polysaccharide layer was selected and transformed into a binary image using the threshold function. Some portion of the boundary of the polysaccharide layer was manually outlined when the thresholding function could not determine where the boundary was. The resulting binary image of the polysaccharide layer was used to identify the centroid and measure distribution of polysaccharide thickness with respect to the centroid. For each sample, five representative images were used to measure the polysaccharide thickness, and the measurements were aggregated to form histograms. The resulting histograms were fitted with Gaussian curves to extract mean and standard deviation of polysaccharide layer thickness.

### In vitro whole blood bactericidal assays

EcN, EcN *ΔkfiC* or EcN iCAP bacterial cultures were grown overnight in LB broth with appropriate antibiotics and IPTG concentrations. The cultures were spun down at 3,000*g* for 5 minutes and resuspended in 1 ml of sterile PBS. They were further normalized to an OD_600_ of 1 with sterile PBS. Next, 150 µl of blood from the single donor human whole blood or murine (BALB/c) whole blood (Innovative Research) was aliquoted into three wells per strain in a 96-well plate. Then, 1.5 µl of bacteria was added to each well and incubated at 37 °C. At various time points, the plate was taken out, and a serial dilution of each sample was prepared in PBS. The dilutions were plated on LB agar plates with erythromycin. The agar plates were incubated at 37 °C overnight and inspected the next day for CFU counting.

### Phagocytosis assays

The phagocytosis assays were performed via protocols as previously reported^68,69^. In brief, BMDMs were thawed on a 15-cm non-TC-treated Petri dish and cultured in RPMI with 10% FBS and MCSF for 4 days before the experiment. On the fourth day, BMDMs were collected, counted and diluted to 2 × 10^5^ cells per milliliter in RPMI with 10% FBS (without antibiotics). Afterwards, 1 ml of the new mixture was plated per well (2 × 10^5^ cells) in a 24-well TC-treated plate and cultured overnight. Media in the 24-well BMDM plate were removed the next day, and 1 ml of EcN iCAP constitutively expressing green fluorescent protein (GFP) with or without IPTG induction resuspended in RPMI with 10% FBS without antibiotics was added into each well at a multiplicity of infection of 100. The co-culture was incubated for 30 minutes at 37 °C, followed by rigorous washing with PBS at least three times. Next, 1 ml of RPMI with 10% FBS and gentamicin (30 µg ml^−1^) was added to each well, followed by live imaging under confocal microscopy. The BMDMs were then lysed with 0.5% Triton X in PBS, and lysates were collected and plated on LB agar with erythromycin, followed by overnight incubation at 37 °C. Colonies were counted the next day. ImageJ was used to count the number of macrophages, engulfed bacterial cells and macrophages containing engulfed bacterial cells from the confocal images. The phagocytic index was calculated according to the following formula: phagocytic index = (total number of engulfed bacterial cells / total number of counted macrophages) × (number of macrophages containing engulfed bacterial cells / total number of counted macrophages) × 100.

### Multiplexed cytokine assay of in vitro co-culture

For in vitro cytokine measurement, we used THP-1 cells (American Type Culture Collection (ATCC) TIB-202) co-cultured with bacteria. THP-1 cells were maintained in RPMI-1640 supplemented with 10% FBS, 2 mM L-glutamine, 100 µg ml^−1^ of streptomycin, 100 µg ml^−1^ of penicillin and 0.1% mercaptoethanol at 37 °C and 5% CO_2_. Cells were passaged every 72 hours and maintained inside a tissue culture incubator at 37 °C and 5% CO_2_. For cell quantification and viability analysis, cells were stained using trypan blue stain. For co-culture, THP-1 cells were resuspended at a concentration of 1 × 10^6^ cells per milliliter in RPMI-1640 supplemented with 10% FBS and 0.1% gentamycin. Next, 300 µl of cell suspension was transferred into each well of a 24-well plate, and 3 µl of each bacterial strain was then added to cell culture wells. Subsequently, the culture medium was harvested and centrifuged at 200*g* for 5 minutes to isolate THP-1 without causing cell death. Supernatant was then centrifuged at 3,000*g* for 5 minutes to remove bacteria. The resulting supernatant was analyzed for cytokine response. Immunology multiplexed assay was performed using Milliplex Human Cytokine/Chemokine panel (HCYTMAG-60K-PX29, EMD Millipore) according to the manufacturer’s instructions at the Biomarkers Core Laboratory in the Irving Institute for Clinical and Translational Research.

### Animal models

All animal experiments were approved by the Institutional Animal Care and Use Committee (Columbia University, protocols ACAAAN8002 and AC-AAAZ4470). For tumor-bearing animals, euthanasia was required when the tumor burden reached 2 cm in diameter or after recommendation by the veterinary staff. Mice were blindly randomized into various groups. Animal experiments were performed on 6–8-week-old female BALB/c mice (Taconic Biosciences) unless otherwise noted. Tumor models were established with bilateral subcutaneous hind flank injection of mouse colorectal carcinoma CT26 cells (ATCC CRL-2638) or mammary fat pad injection of 4T1 luciferase mammary carcinoma cells (Kang, Princeton University). All cells were cultured in RPMI-1640 media (Gibco) supplemented with 10% FBS (Gibco) and 1% penicillin–streptomycin (CellGro) and maintained inside a tissue culture incubator at 37 °C and 5% CO_2_. The concentration for implantation of the tumor cells was 5 × 10^7^ cells per milliliter in RPMI (no phenol red). Cells were injected at a volume of 100 μl per flank, with each implant consisting of 5 × 10^6^ cells. Female transgenic MMTV-PyMT mice (Jackson Laboratory, FVB/N background, 6 weeks old), which develop mammary tumors, were also used. Tumors were grown to an average of approximately 400 mm^3^ before experiments. Tumor volume was quantified using calipers to measure the length, width and height of each tumor (V = L × W × H × 0.5). Because the *z* dimension of PyMT tumor is highly variable, total volume was calculated as length × width^2^ × 0.5. Volumes were normalized to pre-injection values to calculate relative or percent tumor growth on a per-mouse basis.

### Bacterial administration for in vivo experiments

Overnight cultures of EcN, EcN *ΔkfiC* and EcN iCAP were grown in LB medium with the appropriate antibiotics and inducers. A 1:100 dilution in LB with appropriate antibiotics and inducers was made the following day and grown in a 37 °C shaker until OD_600_ = 0.1–0.4 (mid-log phase). Cultures were centrifuged at 3,000*g* for 10 minutes and washed three times with cold sterile PBS. The bacteria were then normalized to a desired OD_600_. Unless otherwise noted, i.v. injections were given through the tail vein at the dose of 5 × 10^6^ cells per milliliter (OD_600_ of 0.5) in PBS with a total volume of 100 µl per mouse. Intratumoral injections of bacteria were performed at a concentration of 5 × 10^6^ cells per milliliter with a total volume of 40 µl per tumor. Intraperitoneal injections were injected at varying concentrations in PBS with a total volume of 100 µl per mouse. For induction of TT production, AHL subcutaneous injection was given to mice daily at 10 µM concentration with a total volume of 500 µl per mouse. For in situ activation of iCAP, water containing 10 mM IPTG was given to mice 1 day after bacterial administration.

### Biodistribution and in vivo animal imaging

All bacterial strains used in this study had integrated *luxCDABE* cassette that could be visualized by IVIS spectrum imaging system (PerkinElmer) and were quantified by Living Image software. Images and body weight of mice were obtained every day starting the day of bacterial administration until the study endpoint. At the study endpoint, mice were euthanatized by carbon dioxide, and the tumors and organs (spleen, liver and lungs) were extracted and imaged. They were later weighed and homogenized using a gentleMACS tissue dissociator (C Tubes, Miltenyi Biotec). Homogenates were serially diluted with sterile PBS and plated on LB agar plates with erythromycin and incubated overnight at 37 °C. Colonies were counted the next day.

### ELISA, cytokine multiplexed assay and blood cell count of in vivo animal model

For in vivo cytokine measurement, mice were injected intravenously with each bacterial strain. Subsequently, blood was collected from the submandibular vein and centrifuged at 2,000*g* for 10 minutes in a refrigerated centrifuge at 4 °C. The resulting supernatant was collected and stored at −80 °C until analysis for cytokine response. TNFα was measured using a Quantikine ELISA Kit (R&D Systems) according to the manufacturer’s protocol, and immunology multiplexed assay was performed using Milliplex Mouse Cytokine/Chemokine panel (MCYTOMAG-70K-PMX, EMD Millipore) according to the manufacturer’s instructions at the Biomarkers Core Laboratory in the Irving Institute for Clinical and Translational Research. Neutrophil counts were measured by performing complete blood count on mouse whole blood.

### Characterization of phage sensitivity of intratumoral bacteria

Mice bearing bilateral subcutaneous CT26 tumors were intravenously administered with each bacterial strain. Intratumoral bacteria was isolated after 2 days from supernatants of the homogenized tumors in 5 ml of PBS (15% glycerol). Next, 1 µl of each supernatant was added to 100 µl of LB in a 96-well plate (Corning) in triplicate for control and phage groups. Then, 1 µl of 10^8^ PFU ΦK1-5 phage or PBS was added to each well appropriately. The samples were incubated at 37 °C with shaking in a Tecan plate reader, and the OD_600_ was measured every 20 minutes.

### Statistical analysis

Statistical tests were performed in either GraphPad Prism 7.0 and 8.0 (Student’s *t*-test and ANOVA) or Microsoft Excel. The details of the statistical tests are indicated in the respective figure legends. When data were approximately normally distributed, values were compared using Student’s *t*-test, one-way ANOVA for single variable or two-way ANOVA for two variables. Mice were randomized into different groups before experiments.

### Reporting Summary

Further information on research design is available in the Nature Research Reporting Summary linked to this article.

## Online content

Any methods, additional references, Nature Research reporting summaries, source data, extended data, supplementary information, acknowledgements, peer review information; details of author contributions and competing interests; and statements of data and code availability are available at 10.1038/s41587-022-01244-y.

## Supplementary information

Supplementary Information Supplementary Figs. 1–24, Tables 1–3, Note and Figure Source Data.

Reporting Summary.

Supplementary Table 3 Relevant gene sequences used in this study.

## Data Availability

All data supporting the results are available in the main text or the supplementary materials. Additional data are available from the corresponding author upon reasonable request. *E. coli* Nissle 1917 genome sequences were obtained from the National Center of Biotechnology Information under accession code NZ_CP022686.1. Source data are provided with this paper.

## Source data

Source Data Fig. 3 Unprocessed gels.

## Code availability

Computational model derivation is described in the Supplementary Note. The MATLAB code used in this study is available from the corresponding author upon reasonable request.
