# Supplementary material for: A programmable encapsulation system improves delivery of therapeutic bacteria in mice
Source: Nat Biotechnol. 2022 Mar 17;40(8):1259–69. doi: 10.1038/s41587-022-01244-y (PMC9371971; doi:10.1038/s41587-022-01244-y)
Supplement: Supplementary file 2 — Reporting Summary. [file 41587_2022_1244_MOESM2_ESM.pdf]

nature research

Corresponding author(s): Tal Danino, Kam Leong

Last updated by author(s): Jan 21, 2022

# Reporting Summary

Nature Research wishes to improve the reproducibility of the work that we publish. This form provides structure for consistency and transparency in reporting. For further information on Nature Research policies, see our Editorial Policies and the Editorial Policy Checklist.

## Statistics

For all statistical analyses, confirm that the following items are present in the figure legend, table legend, main text, or Methods section.

| n/a | Confirmed | |
|---|---|---|
| ☐ | ☒ | The exact sample size (*n*) for each experimental group/condition, given as a discrete number and unit of measurement |
| ☐ | ☒ | A statement on whether measurements were taken from distinct samples or whether the same sample was measured repeatedly |
| ☐ | ☒ | The statistical test(s) used AND whether they are one- or two-sided<br>*Only common tests should be described solely by name; describe more complex techniques in the Methods section.* |
| ☒ | ☐ | A description of all covariates tested |
| ☐ | ☒ | A description of any assumptions or corrections, such as tests of normality and adjustment for multiple comparisons |
| ☐ | ☒ | A full description of the statistical parameters including central tendency (e.g. means) or other basic estimates (e.g. regression coefficient) AND variation (e.g. standard deviation) or associated estimates of uncertainty (e.g. confidence intervals) |
| ☐ | ☒ | For null hypothesis testing, the test statistic (e.g. $F$, $t$, $r$) with confidence intervals, effect sizes, degrees of freedom and $P$ value noted<br>*Give P values as exact values whenever suitable.* |
| ☒ | ☐ | For Bayesian analysis, information on the choice of priors and Markov chain Monte Carlo settings |
| ☒ | ☐ | For hierarchical and complex designs, identification of the appropriate level for tests and full reporting of outcomes |
| ☒ | ☐ | Estimates of effect sizes (e.g. Cohen's *d*, Pearson's *r*), indicating how they were calculated |

*Our web collection on statistics for biologists contains articles on many of the points above.*

## Software and code

Policy information about availability of computer code

| | |
|---|---|
| Data collection | Living Image v4.7.3 was used for collecting in vivo images, Thermo Fisher TEM User Interface v1.15 and TEM Imaging & Analysis were used for acquiring TEM images, i-control v2.0 was used for collecting plate reader data, Image Lab v6.1 was used for acquiring gel images, and EVOS FL Auto 2 v2.0.802.0 was used for collecting microscopy images. |
| Data analysis | Graphpad Prism v7 and v8 were used for general statistical analysis, ImageJ v1.52 for image processing, MATLAB v2020a for TEM image analysis and mathematical modeling, G*Power v3.1 for power analysis. |

For manuscripts utilizing custom algorithms or software that are central to the research but not yet described in published literature, software must be made available to editors and reviewers. We strongly encourage code deposition in a community repository (e.g. GitHub). See the Nature Research guidelines for submitting code & software for further information.

## Data

Policy information about availability of data

All manuscripts must include a data availability statement. This statement should provide the following information, where applicable:
- Accession codes, unique identifiers, or web links for publicly available datasets
- A list of figures that have associated raw data
- A description of any restrictions on data availability

All data supporting the results are available in the manuscript or the supplementary materials. Additional data are available from the corresponding author on request. E. coli Nissle 1917 genome sequences were obtained from NCBI accession code NZ_CP022686.1.

# Field-specific reporting

Please select the one below that is the best fit for your research. If you are not sure, read the appropriate sections before making your selection.

☒ Life sciences ☐ Behavioural & social sciences ☐ Ecological, evolutionary & environmental sciences

For a reference copy of the document with all sections, see nature.com/documents/nr-reporting-summary-flat.pdf

# Life sciences study design

All studies must disclose on these points even when the disclosure is negative.

| | |
|---|---|
| Sample size | We calculated sample size in our studies by using a power analysis and appropriate statistical test in G*Power 3.1 software. Previous animal studies, or small pilot studies when necessary, served as the basis for calculations of expected averages and deviations used to calculate power, for which we set experiment studies to a value of 0.8. This typically resulted in an experimental group size to be n = 4–7, depending on the experiment. Sample size is explicitly stated for each experimental group for individual experiments in figure captions and data descriptions. |
| Data exclusions | No data excluded. |
| Replication | All experiments were performed at least with triplicates unless otherwise stated. The replication numbers of each data is indicated in the figure legends. In vivo efficacy, safety, and translocation experiments were successfully repeated using CT26 model. |
| Randomization | Cells were randomly allocated into experimental groups. Animals were randomly allocated into experimental groups from a pool of animals with comparable tumor volume at the beginning of all experiments. |
| Blinding | For in vitro experiments, investigators were not blinded because the nature of quantitative analysis is not subject to human bias. Animals were treated by blinded independent researcher. |

# Reporting for specific materials, systems and methods

We require information from authors about some types of materials, experimental systems and methods used in many studies. Here, indicate whether each material, system or method listed is relevant to your study. If you are not sure if a list item applies to your research, read the appropriate section before selecting a response.

## Materials & experimental systems

| n/a | Involved in the study | |
|---|---|---|
| ☒ | ☐ | Antibodies |
| ☐ | ☒ | Eukaryotic cell lines |
| ☒ | ☐ | Palaeontology and archaeology |
| ☐ | ☒ | Animals and other organisms |
| ☒ | ☐ | Human research participants |
| ☒ | ☐ | Clinical data |
| ☒ | ☐ | Dual use research of concern |

## Methods

| n/a | Involved in the study | |
|---|---|---|
| ☒ | ☐ | ChIP-seq |
| ☒ | ☐ | Flow cytometry |
| ☒ | ☐ | MRI-based neuroimaging |

## Eukaryotic cell lines

Policy information about cell lines

| | |
|---|---|
| Cell line source(s) | THP-1 and CT26 cell lines were obtained from ATCC. 4T1 luciferase cell line was obtained from Yibin Kang from Princeton University. |
| Authentication | Cell lines purchased from ATCC were frozen at early passage, thus did not require additional authentication. 4T1 cells were authenticated based on the morphology and growth, as well as their ability to form orthotopic tumors in vivo. |
| Mycoplasma contamination | All cell lines tested negative for mycoplsma contamination. |
| Commonly misidentified lines (See ICLAC register) | No commonly misidentified cell lines were used. |

## Animals and other organisms

Policy information about studies involving animals; ARRIVE guidelines recommended for reporting animal research

| | |
|---|---|
| Laboratory animals | 6–8 weeks old BALB/c females were purchased from Taconic Biosciences for in vivo experiments. PyMT-MMTV model (FVB/N, 6 |

| | |
|---|---|
| Laboratory animals | weeks old, females) was purchased from Jackson Laboratory and bred in house. Mice were housed in a BSL-2 compatible, SPF facility in negative pressure vented cages. The room is fully air-conditioned at 20-26 °C at 30-70% humidity with a 12-hour dark light cycles. |
| Wild animals | This study did not involve wild animals. |
| Field-collected samples | This study did not involve samples collected from the field. |
| Ethics oversight | All animal experiments were approved by the Institutional Animal Care and Use Committee (Columbia University, protocols ACAAAN8002 and AC-AAAZ4470). |

Note that full information on the approval of the study protocol must also be provided in the manuscript.
