# Supplementary material for: A programmable encapsulation system improves delivery of therapeutic bacteria in mice
Source: Nat Biotechnol. 2022 Mar 17;40(8):1259–69. doi: 10.1038/s41587-022-01244-y (PMC9371971; doi:10.1038/s41587-022-01244-y)
Supplement: Source Data Fig. 3 — Unprocessed gels. [file 41587_2022_1244_MOESM4_ESM.pdf]

Figure Source Data

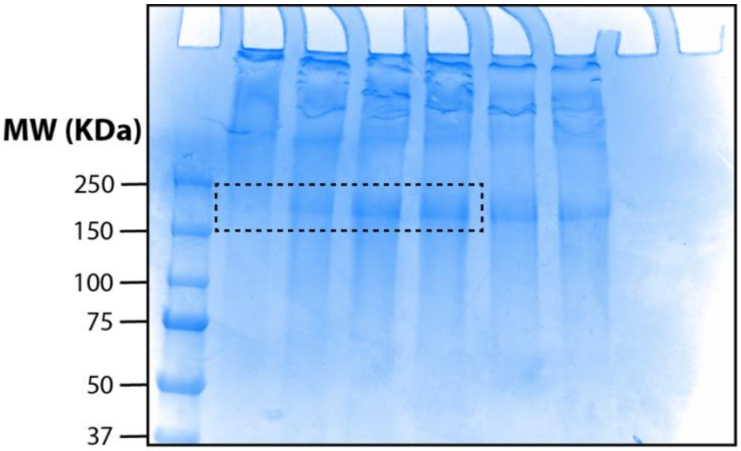

Uncropped SDS-PAGE image of Figure 3b. Dotted black box indicates cropped area for Figure 3b.

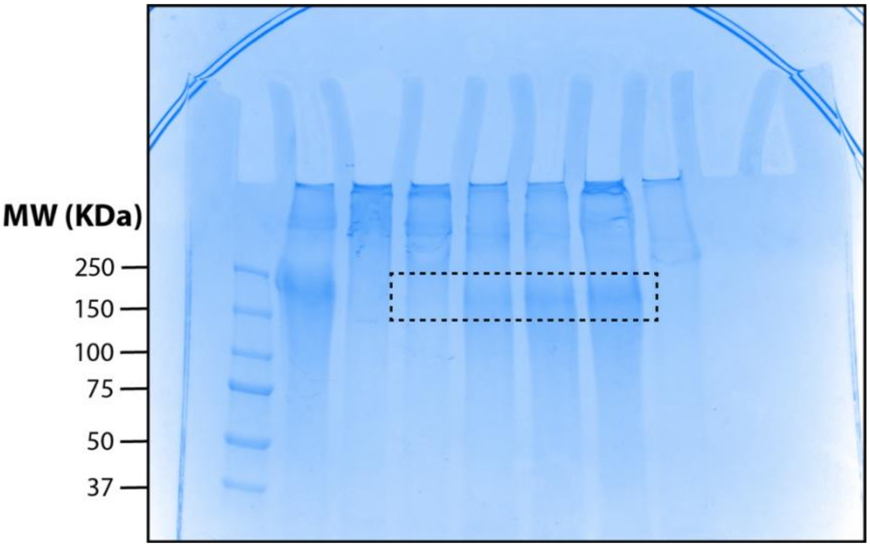

Uncropped SDS-PAGE image of Figure 3c (left). Dotted black box indicates cropped area for Figure 3c (left).

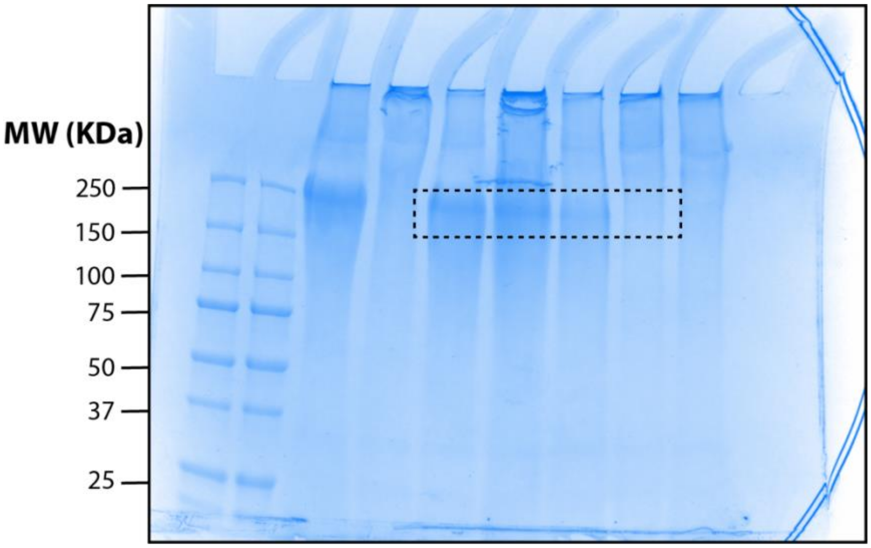

Uncropped SDS-PAGE image of Figure 3c (right). Dotted black box indicates cropped area for Figure 3c (right).
